# Ecological Niche Characterization and Potential Distribution of the Subendemic Species *Cicer grande* (Popov) Korotkova (Fabaceae) in Uzbekistan

**DOI:** 10.3390/plants15172727

**Published:** 2026-09-06

**Authors:** Bekzod Mavlanov, Xuexi Ma, Khusniddin Abulfayzov, Khabibullo Shomurodov, Natalya Beshko, Azizbek Abduraimov, Azizbek Maxmudov, Ozodbek Abduraimov, Xi Chen, Yaoming Li

**Affiliations:** 1 Institute of Botany Academy Sciences Republic of Uzbekistan, Tashkent 100125, Uzbekistan; mavlanov.bekzod@mail.ru (B.M.); husniddin.abulfayzov1@mail.ru (K.A.); h.shomurodov@mail.ru (K.S.); natalia_beshko@mail.ru (N.B.); ozodbek88@bk.ru (O.A.); 2 Key Laboratory of Ecological Safety and Sustainable Development in Arid Lands, Xinjiang Institute of Ecology and Geography, Chinese Academy of Sciences, Urumqi 830011, China; chenxi@zjut.edu.cn (X.C.); lym@ms.xjb.ac.cn (Y.L.); 3 Research Center for Ecology and Environment of Central Asia, Chinese Academy of Sciences, Urumqi 830011, China; 4 Department medicinal plants and botany, Gulistan State University, Gulistan 120100, Uzbekistan; abduraimov2017@inbox.ru; 5 College of Geoinformatics, Zhejiang University of Technology, Hangzhou 310014, China

**Keywords:** *Cicer grande*, ecological niche, species distribution modeling, subendemic species, Uzbekistan

## Abstract

*Cicer grande* (Popov) Korotkova is a rare, subendemic species within the flora of Uzbekistan, primarily inhabiting mountainous landscapes. As a wild progenitor of nutritionally significant cultivated crops, it holds considerable potential for enhancing food security and mitigating hunger through applications in sustainable agriculture and the conservation of plant genetic resources. This study employed species distribution modeling to examine the ecological niche, geographic distribution, and potential habitat suitability of *C. grande* across Uzbekistan. Data from herbarium specimens and field surveys were integrated with a comprehensive suite of environmental variables encompassing climatic, edaphic, topographic, hydrological, and anthropogenic factors. From an initial set of 45 predictor variables, highly correlated ones were removed using Pearson correlation analysis and variance inflation factor (VIF) assessment to reduce multicollinearity. Habitat suitability was subsequently modeled using the BIOCLIM algorithm. The results show that *C. grande* is predominantly found at elevations between 1000 and 2500 m, where it is strongly associated with shallow Lithosols and carbonate-rich Calcic Xerosols (FAO soil classification). Phytocoenotic assessments indicate that the species occurs mainly within plant communities dominated by perennial species. Among the environmental drivers analyzed, elevation, temperature-related variables, and slope aspect were identified as the most influential factors shaping its distribution. BIOCLIM projections reveal that highly suitable habitats are spatially limited and largely confined to the Nurata and Kuhitang mountain ranges. Overall, these findings demonstrate that *C. grande* occupies a narrow and specialized ecological niche, which likely accounts for its restricted distribution and subendemic status. This study provides a robust scientific foundation for developing conservation strategies and habitat management plans for this rare species in Central Asia.

## 1. Introduction

Plant biodiversity is a cornerstone of terrestrial ecosystems, underpinning their functioning, stability, and provision of vital services for human well-being [1]. Its profound link to human societies highlights its essential role in sustaining livelihoods and ecological resilience [2]. Yet, this diversity is facing unprecedented global decline, with extinction risks escalating across biomes due to human-driven pressures such as habitat loss, overexploitation, pollution, and climate change [3,4]. These pressures lead to habitat fragmentation, population decline, and extinction, disproportionately affecting species with narrow ranges and ecological tolerances, many of which are endemic or subendemic (i.e., largely restricted to a specific region but extending marginally into adjacent areas) and highly sensitive to environmental change [5,6]. This erosion of plant diversity constitutes a major challenge for ecology and conservation [7].

In response, international frameworks like those of the IUCN have developed standardized criteria for assessing extinction risk and guiding conservation priorities [8,9]. However, critical knowledge gaps persist at regional and local scales regarding the distribution, ecology, and viability of many species, limiting effective action [10,11]. While protected areas are central to conservation strategies, their success hinges on accurately capturing species’ ecological niches and distributions, which are shaped by complex climatic, edaphic, topographic, and anthropogenic interactions [12,13]. Therefore, precisely defining the ecological niches and habitat suitability of threatened species is fundamental for evidence-based conservation planning [14].

This need is particularly acute in globally significant biodiversity hotspots like the mountains of Central Asia. This region, situated at the junction of Mediterranean and Asian floristic zones and characterized by complex topography, hosts about 5000–6000 vascular plant species with remarkably high endemism (~25%) [15,16,17]. Uzbekistan, with its diverse landscapes and climates, is a key area for studying such distribution patterns. Research here increasingly focuses on species of economic and nutritional importance to support food security and sustainable management [18,19,20]. Studying regionally restricted taxa such as *C. grande* is thus of great ecological, biogeographical, and conservation significance. Advances in species distribution modeling (SDM), supported by high-resolution environmental datasets (e.g., WorldClim, TerraClimate, SRTM), now enable robust assessments of ecological niches and potential distributions [21,22].

The genus *Cicer* L. (Fabaceae), comprising 44 species primarily distributed across Central and Southwest Asia, the Mediterranean, and East Africa, includes 43 wild species that serve as a crucial genetic reservoir for crop improvement and climate resilience [23,24,25]. Against this background, the present study aims to: (i) characterize the ecological niche of *C. grande*, (ii) model its current and potential distribution in Uzbekistan, and (iii) identify the key environmental factors shaping its distribution. By integrating ecological modeling with conservation science, this work seeks to advance the understanding of plant distribution in arid mountain ecosystems and provide a scientific basis for conserving Central Asia’s unique plant diversity.

## 2. Results

### 2.1. Ecological Characteristics and Phytocoenotic Affinity of C. grande

#### 2.1.1. Morphological Traits and Phenological Characteristics

*C. grande* is a perennial herbaceous species reaching 20–25 cm in height, characterized by a twisted stem densely covered with pubescence. The leaves are 4–7 cm long, with petioles terminating either in a simple apex or bearing 3–5 tendrils. Leaflets occur in 4–7 pairs and are oblong-elliptic in shape, measuring 10–25 mm in length and 6–12 mm in width. Both adaxial and abaxial surfaces of the leaflets are covered with short glandular hairs, contributing to the species’ distinctive indumentum. The peduncle measures 2.5–3.5 cm in length, while the pedicel is 4–6 mm long. The calyx is approximately 10 mm in length, and the ovary is densely pubescent. These morphological features collectively distinguish *C. grande* from closely related taxa within the genus. One of the most important and distinctive characteristics of representatives of the genus *Cicer* is their role as wild progenitors and genetic relatives of cultivated chickpea, possessing valuable adaptive traits and enhanced tolerance to a wide range of environmental stresses [26,27]. 

#### 2.1.2. Global Distribution Patterns of the Genus *Cicer* L.

**Figure 1:**
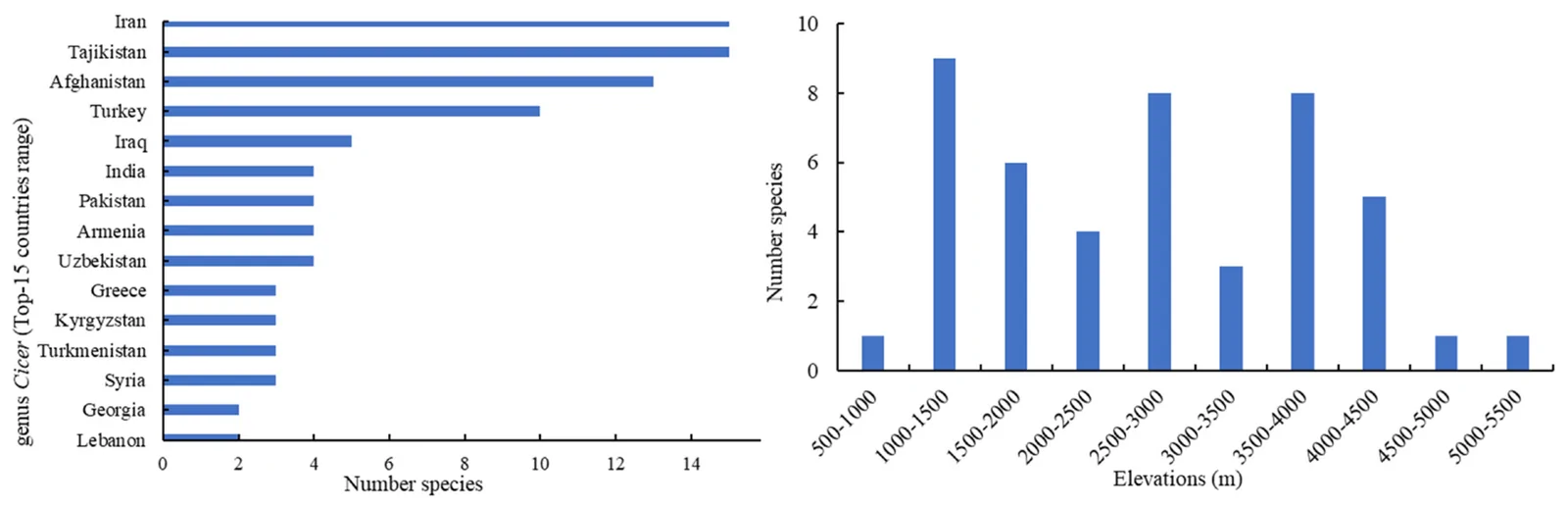
Geographic distribution and elevational range of species within the genus *Cicer* L.

Based on available data, species of the genus *Cicer* exhibit a broad geographical distribution across a wide range of biogeographic regions. The genus is primarily distributed in Western Asia, Central Asia, the Caucasus, the Mediterranean Basin, and the mountainous regions of North Africa (Figure 1).

This distribution pattern reflects the ecological plasticity and evolutionary diversification of the genus across heterogeneous environments. While some *Cicer* species display wide geographic ranges, others are restricted to narrowly defined areas and exhibit pronounced endemic characteristics. Such variation in range size suggests differing ecological tolerances and evolutionary histories among species within the genus, underscoring the importance of regional-scale assessments for understanding patterns of diversity and endemism.

Species richness of the genus *Cicer* is relatively high in Afghanistan, Iran, Turkey, and Tajikistan, indicating these regions as major centers of diversity. Additional occurrences have been documented in South and East Asia, including northern India (Kashmir), Nepal, Pakistan, and northern China, where several species occur at lower frequencies. In the Mediterranean region, wild *Cicer* species have been recorded in Greece, the Balkan countries, Spain, and Morocco, further highlighting the broad biogeographical extent of the genus. Within Central Asia, particularly in Uzbekistan, Kyrgyzstan, and Kazakhstan, multiple *Cicer* species have been identified as part of the regional flora, including *C. songaricum* Stephan ex DC., *C. grande*, and *C. flexuosum* Lipsky. These findings emphasize the ecological and biogeographical significance of Central Asia as an important distribution zone for the genus.

Elevational analyses revealed that *Cicer* species occupy a wide elevational range, extending from lowland areas near sea level to high-mountain environments. Species occurrences were recorded across elevation bands of 0–500 m, 500–2000 m, and 2000–4000 m a.s.l., with certain taxa occurring under extreme alpine conditions at elevations reaching 4600–5600 m. This broad elevational distribution reflects considerable ecological adaptability and tolerance to diverse climatic and environmental conditions.

#### 2.1.3. Plant Community Composition and Phytocenotic Structure

*C. grande* is classified as a rare species within the flora of Uzbekistan and is considered a subendemic taxon of the Western Pamir-Alay region. The species is included in the most recent edition of the Red Data Book of the Republic of Uzbekistan with conservation status category 1 (critically endangered). It inhabits rocky and fine-grained skeletal soils on mid-mountain slopes and reproduces exclusively through seeds, indicating limited regenerative capacity under natural conditions. During the present study, plant communities containing *C. grande* were investigated in the Nurata and Kuhitang mountain ranges [28]. In the Nurata Range, mixed herbaceous–grass–*Artemisia* phytocenoses were predominant. Within this area, the species was primarily recorded on brown, rocky soils. The total vegetation cover reached 70–80%, and a total of 32 vascular plant species were identified within the studied plots. In contrast, the Kuhitang Range was characterized by mixed herbaceous–*Ferula*-dominated phytocenoses. Here, the distribution of *Cicer grande* was mainly associated with fine rocky substrates, and the overall vegetation cover was comparatively lower, reaching approximately 45%. A total of 28 plant species were recorded within the research sites (Table 1).

#### 2.1.4. Population Demography, Ontogenetic Structure, and Edaphic Associations

Across both mountain systems, variations in the floristic composition and vegetation cover of the phytocenoses were strongly influenced by soil type and geomorphological features, highlighting the ecological specificity and habitat sensitivity of *C. grande* populations.

The family Asteraceae represented the dominant taxonomic group within the floristic composition, comprising 14.3% of the total recorded species. The Nurata and Kuhitang mountain ranges exhibited comparable levels of species richness; however, a detailed comparative analysis revealed noticeable differences in floristic composition, indicating spatial variation in species assemblages likely driven by local ecological and edaphic conditions.

Initially, the biometric parameters of *C. grande* tussocks within the studied populations were analyzed. The measured traits included total height, aboveground height, root length and diameter, and leaf length and width. Comparison between the two populations (Nurata—LP 1 and Kuhitang—LP 2) revealed that biometric parameters were slightly higher in the Nurata population. Specifically, the total tussock height in the Nurata region ranged 60–63 cm, while in Kuhitang it ranged 43–45 cm. Demographic analysis revealed that tussock numbers per population ranged from 32 to 40, with density per 1 m^2^ ranging from 1.6 to 2 tussocks, and ecological density ranging from 2 to 2.66 tussocks per m^2^. LP 1 corresponds to the mature ontogenetic type, while LP 2 corresponds to the young ontogenetic type. Overall, the populations appear relatively stable (Table 2).

**Figure 2:**
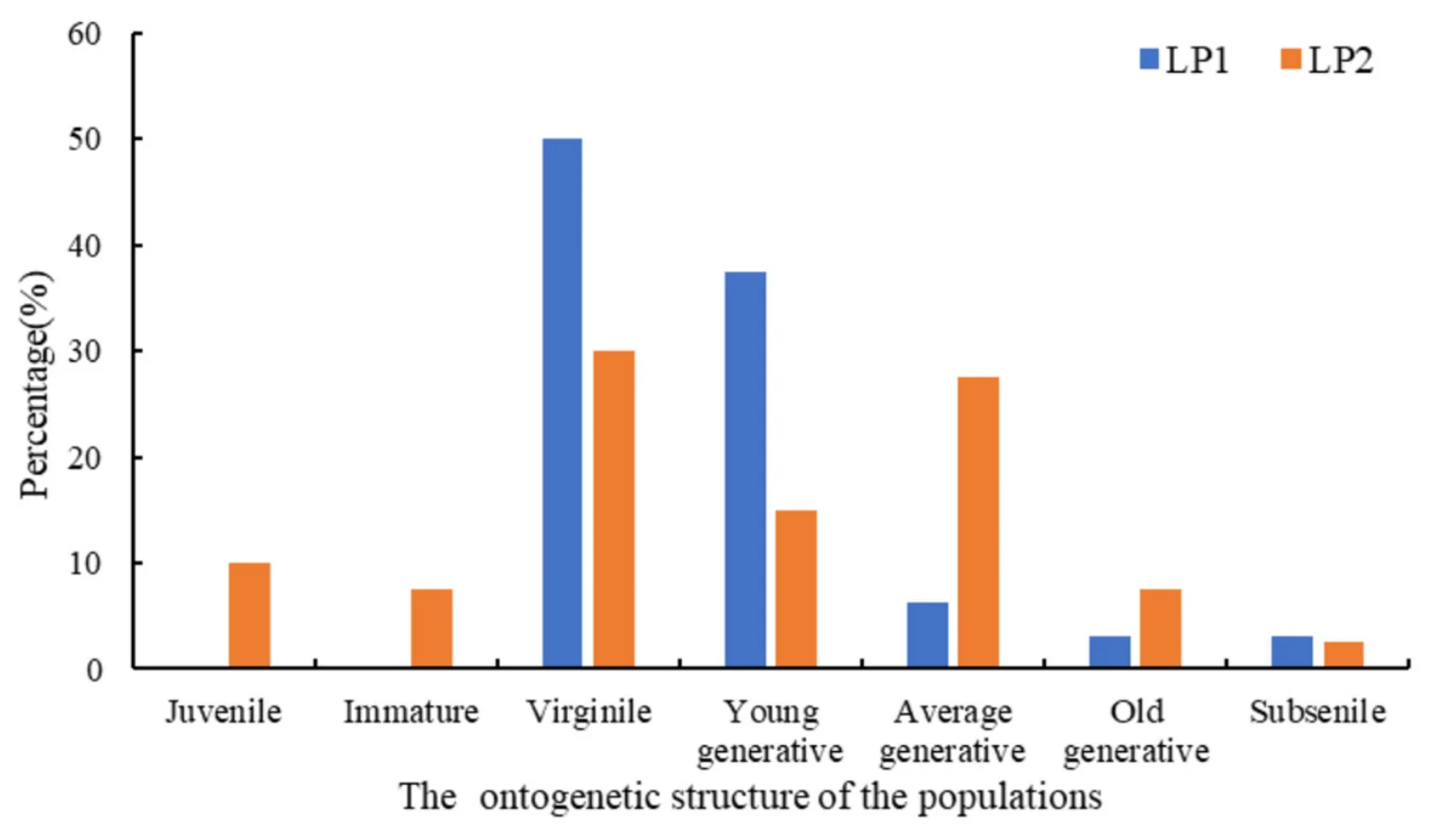
Ontogenetic structure of subpopulations.

The ontogenetic structure of the populations was also analyzed. The Nurata population (LP 1) exhibits a left-skewed ontogenetic spectrum, with virginile and young generative tussocks comprising the majority, accounting for 87.5% of total population abundance. In contrast, the Kuhitang population (LP 2) exhibits a bimodal ontogenetic spectrum, with virginile and middle generative tussocks being dominant (Figure 2).

**Figure 3:**
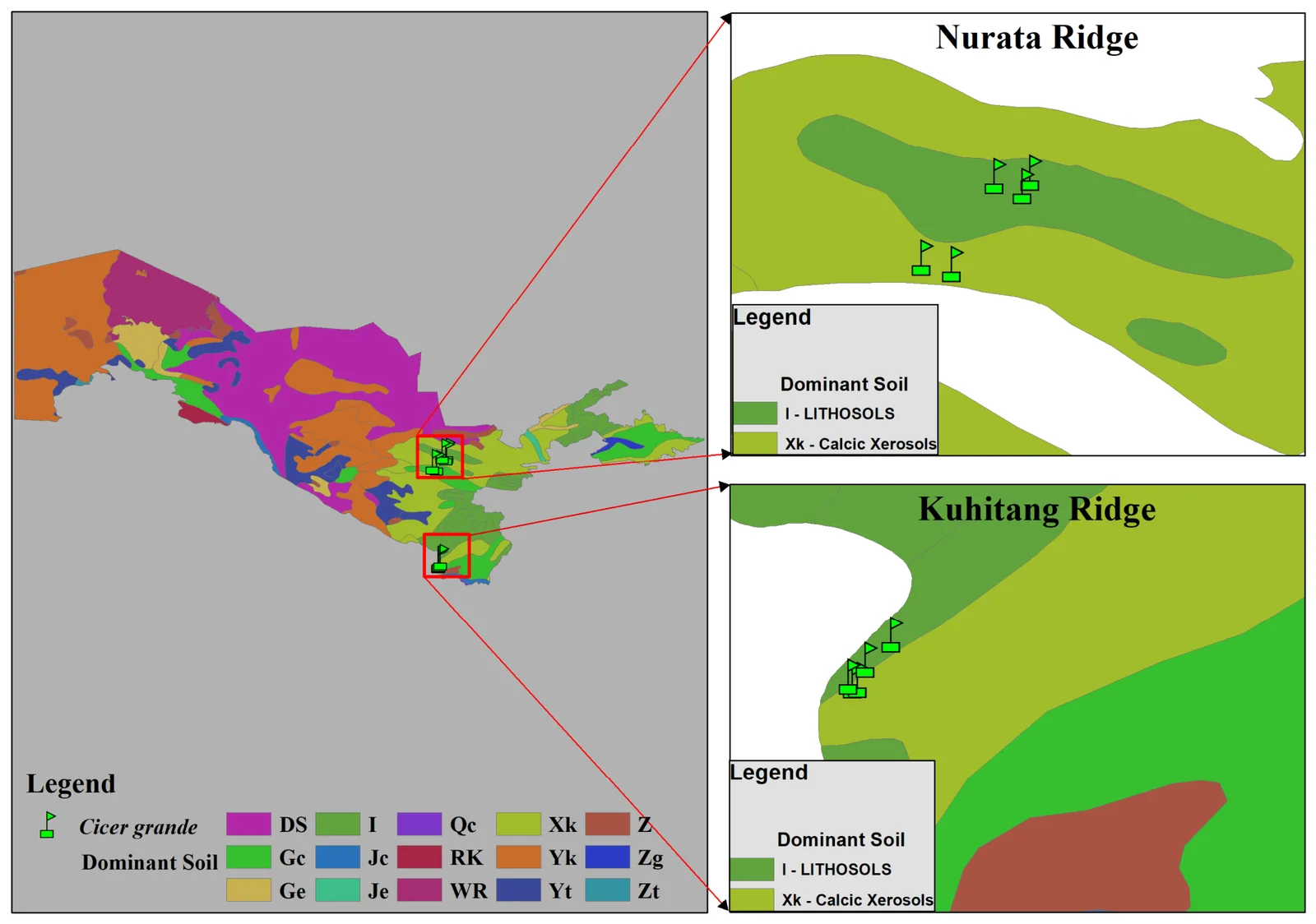
Dominant soil types and occurrence of *C. grande*. I—Lithosols; Xx—Calcic Xerosols.

*C. grande* is a plant species endemic to the mountainous and submontane regions of Uzbekistan, characterized by a restricted distribution range. The results of this study indicate that its primary natural range is closely associated with the Nurata and Kuhitang mountain systems. In both regions, the occurrence of *C. grande* was predominantly correlated with Lithosols and Calcic Xerosols soil types. Lithosols are shallow (less than 30 cm thick), stony soils formed on hard bedrock along mountain slopes, whereas Calcic Xerosols are typical of arid and semi-arid environments and develop over carbonate-rich layers. In Uzbekistan, natural populations of *C. grande* have also been recorded on the slopes of the Chatkal, Kurama, Zarafshon, and Nurata mountain systems. The species’ distribution predominantly occurs between 1000 and 2500 m above sea level, corresponding closely with areas dominated by Lithosols and Calcic Xerosols, reflecting the species’ specific edaphic and ecological requirements (Figure 3).

These results indicate that the distribution of *C. grande* is spatially closely associated with Lithosols and Calcic Xerosols. The aggregation of its occurrence specifically on these soil types clearly highlights the critical role of edaphic factors in shaping the species’ spatial distribution.

### 2.2. Relationship Between Species Richness and Climatic Gradients

**Figure 4:**
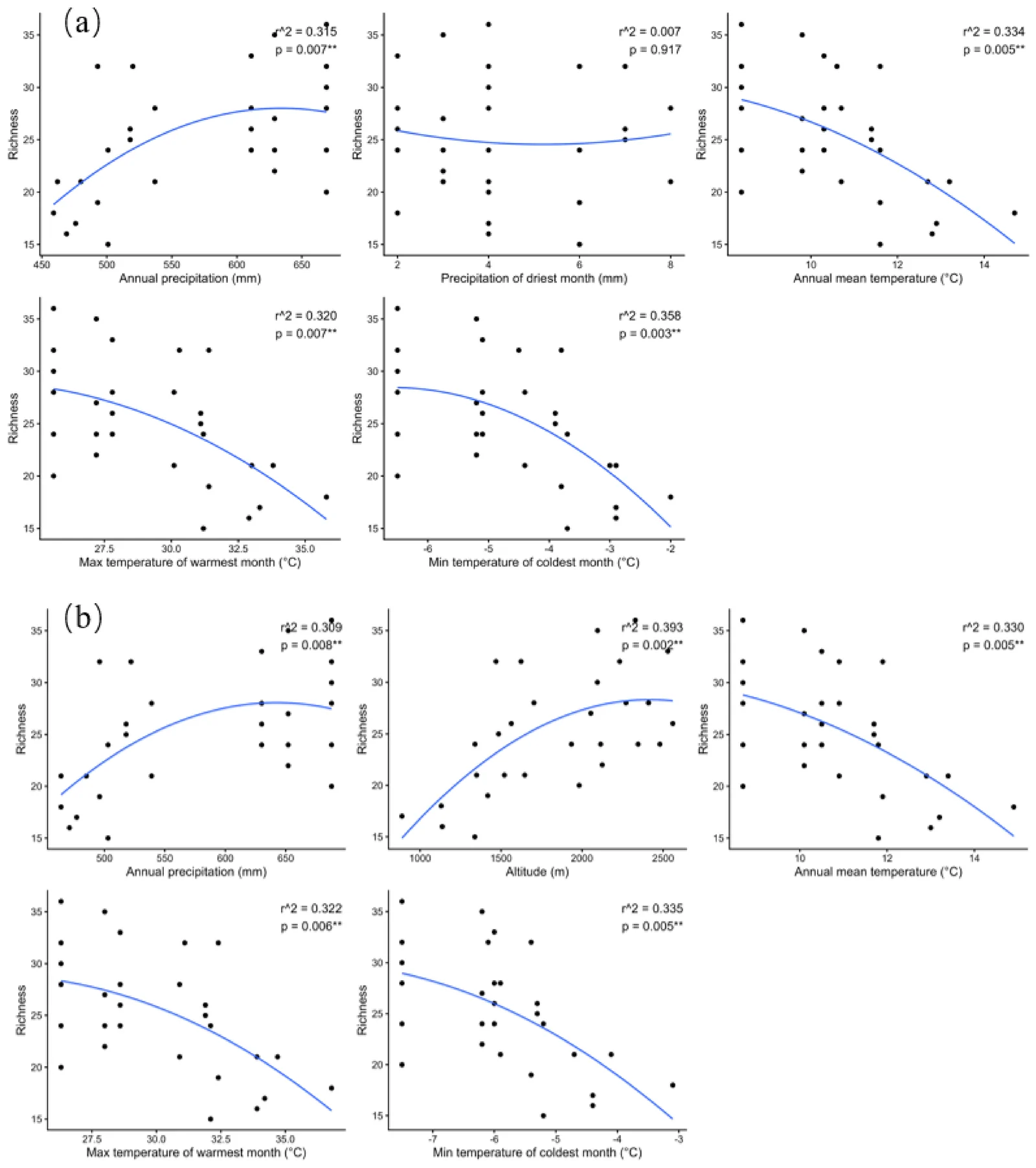
Relationship between species richness and key climatic and geographical factors ((**a**)—Nurata ridge, (**b**)—Kuhitang ridge); ** indicates a statistically significant relationship at *p* < 0.01.

To assess the role of climatic factors in shaping the distribution of *C. grande*, we examined the relationships between species richness and key environmental variables, including annual precipitation, elevation, mean annual temperature, maximum temperature of the warmest month, and minimum temperature of the coldest month (Figure 4). Analyses conducted along the Nurata (A) and Kuhitang (B) transects revealed statistically significant correlations between species richness and these climatic and geographic variables in both regions (Nurata: *p* < 0.01; Kuhitang: *p* < 0.05).

A nonlinear association was observed between annual precipitation and species richness (Nurata: r^2^ = 0.315; Kuhitang: r^2^ = 0.308). Elevation emerged as the strongest predictor, accounting for the largest proportion of variation in species richness across both transects (r^2^ = 0.393). Additionally, increasing mean annual temperature was associated with a decline in species richness in both regions (Nurata: r^2^ = 0.334; Kuhitang: r^2^ = 0.336), indicating a negative effect of warmer conditions on the local occurrence of *C. grande*.

Moreover, a negative correlation was observed between species richness and both the maximum temperature of the warmest month (Nurata: r^2^ = 0.320; Kuhitang: r^2^ = 0.315) and the minimum temperature of the coldest month (Nurata: r^2^ = 0.358; Kuhitang: r^2^ = 0.343). These results indicate a consistent variation in species richness along climatic and elevational gradients across the Nurata and Kuhitang transects. The similar trends observed for the selected variables confirm the presence of overarching ecological patterns between the two regions.

### 2.3. Principal Component Analysis of Habitat Environmental Variables

Analysis of the distribution of *Cicer grande* based on Principal Component Analysis (PCA) indicated that PC1 explained 56.6% of the ecological variation and was primarily associated with variables including bio_2, LST_Night, SRAD, SOC_Proxy, and NPP. PC2 accounted for 24.5% of the variation and was characterized mainly by HumanModification, AlbedoSW, and WindSpeed. Together, PC1 and PC2 explained 81% of the total ecological variation.

**Figure 5:**
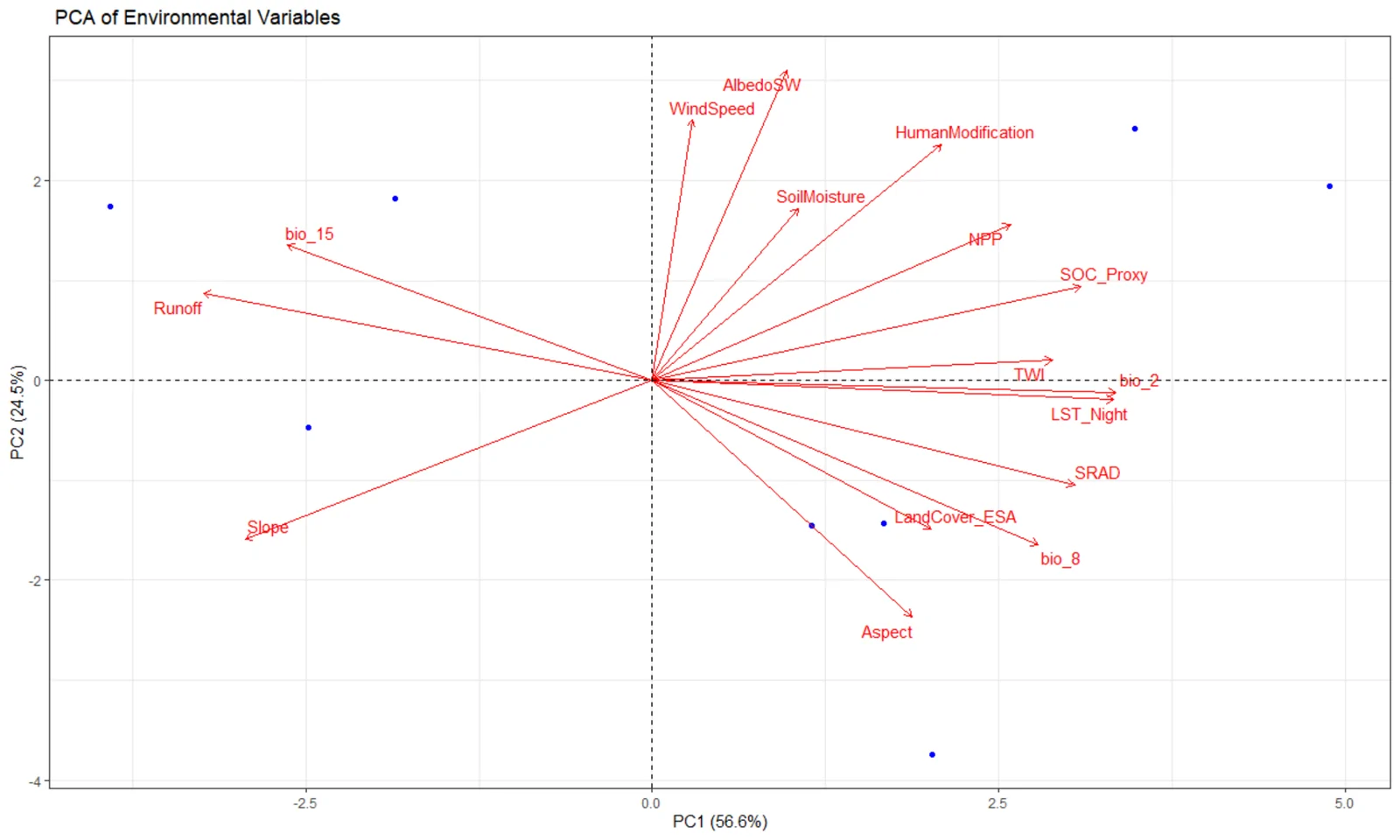
Principal component analysis (PCA) of environmental conditions associated with the occurrence localities of *C. grande*. Points represent PCA scores of the occurrence localities based on environmental values extracted at their geographic coordinates.

Loadings analysis revealed that bio_2, LST_Night, SRAD, and SOC_Proxy had the highest loadings on PC1, whereas HumanModification and AlbedoSW dominated PC2. The PC3 component, associated with SoilMoisture, NPP, and Runoff, accounted for a relatively small proportion of ecological variation. PCA scores indicated that positive PC1 values corresponded to areas with high bio_2, LST_Night, and SRAD, while negative values were associated with low NPP and higher Slope and Runoff. For PC2, positive scores were linked to regions with high anthropogenic influence, whereas negative scores reflected relatively undisturbed environments (Figure 5).

The PCA results showed distinct clustering of ecological variables along primary environmental gradients, highlighting the main factors structuring *C. grande* habitat. This approach facilitated the identification of key distribution drivers, offering essential understanding of the environmental determinants of spatial variability.

### 2.4. Ensemble Modelling of Habitat Suitability and Potential Distribution

#### 2.4.1. Model Evaluation and Algorithm Performance

**Figure 6:**
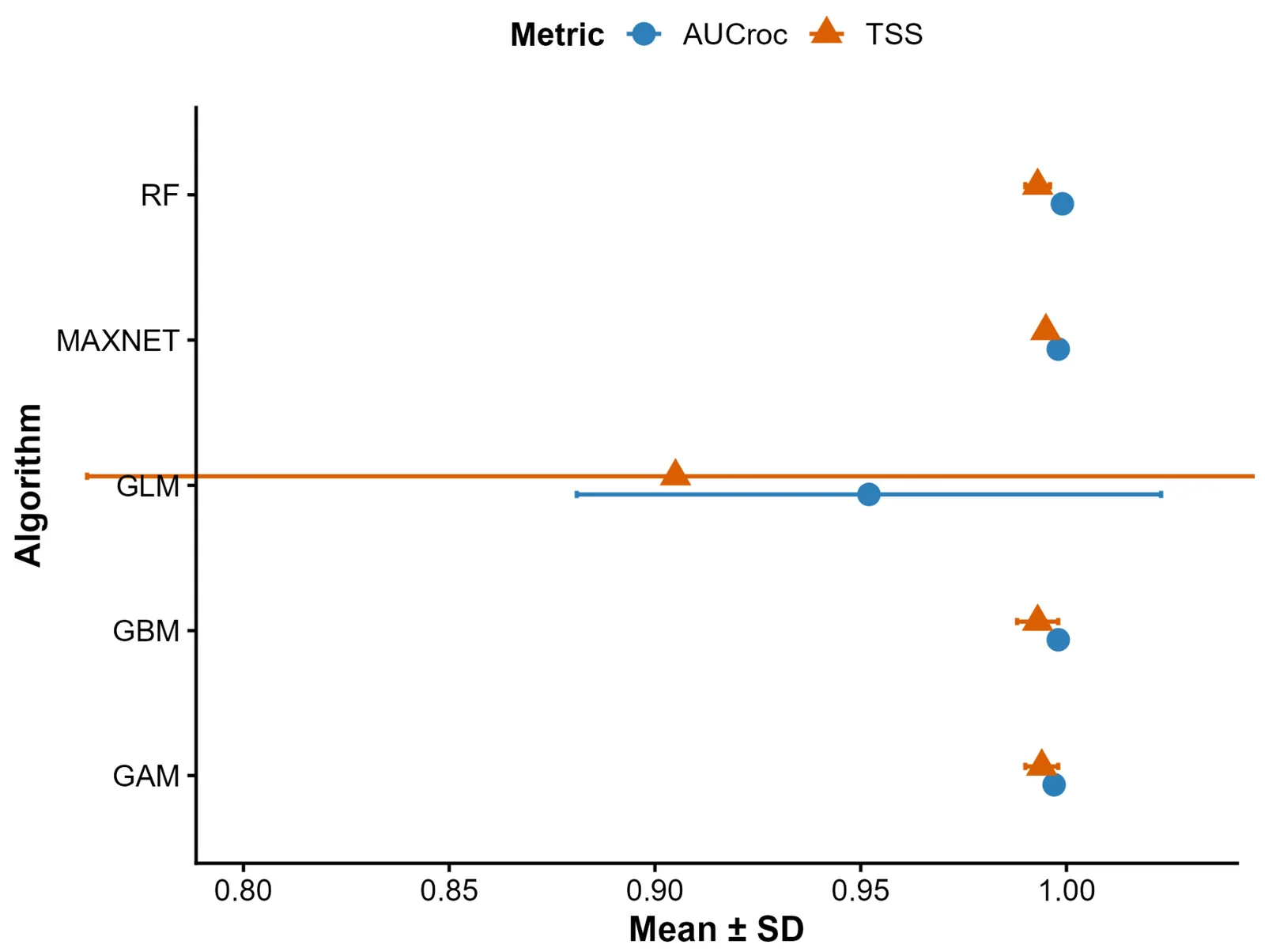
Comparative performance of species distribution models based on AUCcv and TSS metrics.

The five-fold cross-validation showed variable predictive performance among the five modelling algorithms. MAXNET and RF demonstrated the highest cross-validation AUC values, with mean AUC scores of 0.993 ± 0.007 and 0.987 ± 0.020, respectively. GBM showed an intermediate AUC performance (0.959 ± 0.078), whereas GAM and GLM produced lower mean AUC values of 0.873 ± 0.118 and 0.869 ± 0.133, respectively. Based on TSS, MAXNET achieved the highest mean score (0.903 ± 0.128), followed by GBM (0.750 ± 0.197), GAM (0.723 ± 0.258), GLM (0.715 ± 0.287), and RF (0.598 ± 0.259) (Figure 6).

Evaluation using the independent testing dataset showed consistently high predictive performance for most algorithms. The mean independent-test AUC values were 0.952 ± 0.071 for GLM, 0.997 ± 0.002 for GAM, 0.998 ± 0.001 for GBM, 0.999 ± 0.001 for RF, and 0.998 ± 0.000 for MAXNET. Corresponding TSS values were 0.905 ± 0.143, 0.994 ± 0.004, 0.993 ± 0.005, 0.993 ± 0.003, and 0.995 ± 0.001, respectively.

#### 2.4.2. Relative Importance of Environmental Predictors

**Figure 7:**
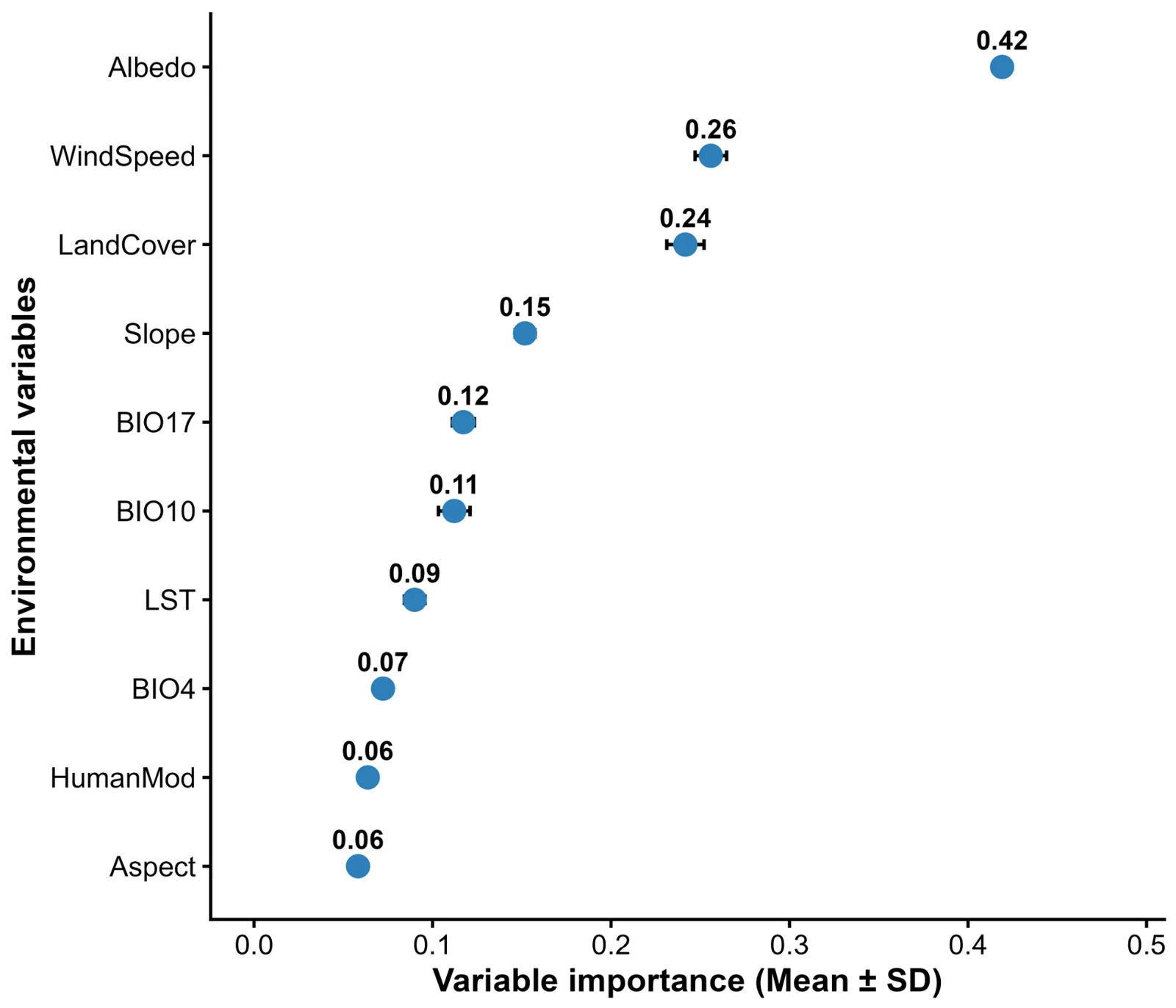
Relative importance of environmental predictors influencing the potential distribution of *C. grande*.

The ensemble model identified substantial differences in the relative importance of the environmental predictors influencing the potential distribution of *C. grande* (Figure 7). Albedo was the most influential predictor, with a mean variable importance of 0.42, followed by Wind Speed (0.26) and Land Cover (0.24). Slope also showed a relatively high contribution (0.15), whereas BIO17 and BIO10 had intermediate importance values of 0.12 and 0.11, respectively. Land Surface Temperature (LST) contributed 0.09, while BIO4 and Human Modification showed lower importance values of 0.07 and 0.06, respectively. Aspect had the lowest relative importance among the selected predictors (0.06).

Overall, the results indicate that surface characteristics, particularly Albedo, together with Wind Speed, Land Cover, and Slope, were the most influential environmental factors determining the predicted distribution of *C. grande*. In contrast, the relatively lower contributions of BIO4, Human Modification, and Aspect suggest that these variables had a weaker influence on the final ensemble predictions (Figure 7).

#### 2.4.3. Response Curves of Key Environmental Predictors

Response curves illustrated the relationships between predicted habitat suitability for *C. grande* and individual environmental predictors, showing how suitability changed along the corresponding environmental gradients. The curves were derived from the AUC-based ensemble model and therefore represent the integrated response of the selected modelling algorithms.

Albedo showed the strongest response among the environmental predictors. Habitat suitability was relatively high at low albedo values and declined sharply as albedo increased, reaching a relatively stable low level at intermediate-to-high values. This pattern is consistent with the high relative importance of albedo identified by the variable-importance analysis.

The response to BIO10 (mean temperature of the warmest quarter) showed a gradual decline in predicted suitability from lower values toward the intermediate temperature range, followed by relatively stable suitability at higher values. BIO17 (precipitation of the driest quarter) showed a similar decreasing response at lower-to-intermediate values, after which predicted suitability remained relatively stable. These patterns suggest that the species’ potential distribution is associated with specific climatic conditions during the warmest and driest periods of the year.

Land Cover also showed a noticeable response, with predicted suitability decreasing from lower land-cover values toward intermediate values and subsequently remaining relatively stable. At the upper end of the gradient, a slight increase in suitability was observed. This pattern indicates that land-cover characteristics contribute to the spatial variation in habitat suitability.

For LST, predicted suitability remained relatively stable across lower and intermediate values but increased gradually toward the upper part of the observed gradient. This suggests a positive response to relatively warmer surface conditions at the upper end of the environmental range.

**Figure 8:**
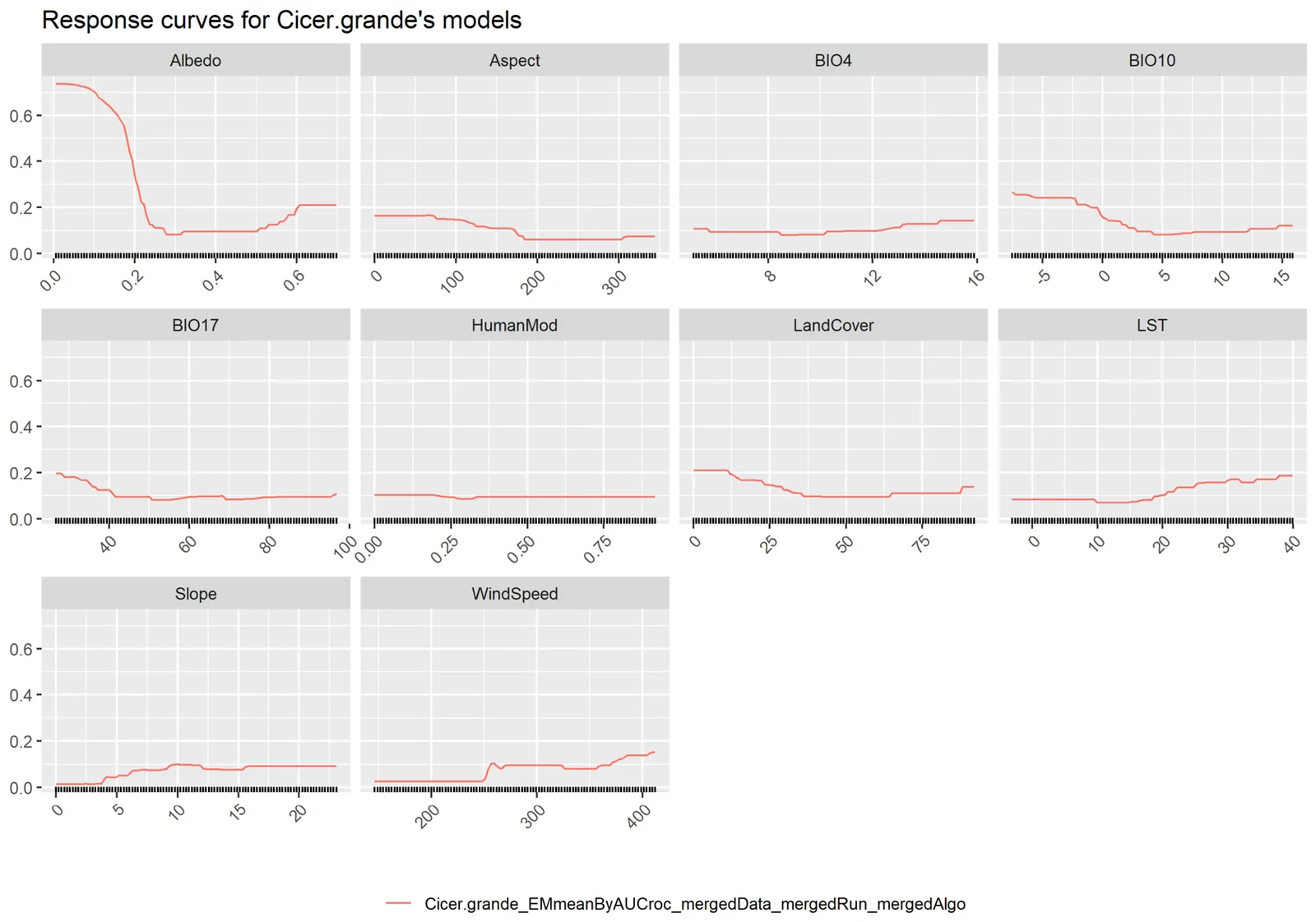
Response curves of environmental predictors showing their effects on the predicted habitat suitability of *C. grande*.

The response to Slope was relatively weak at low values but increased progressively at moderate slope values, after which predicted suitability remained comparatively stable. This indicates that slope contributes to habitat differentiation, although its overall importance was lower than that of albedo, wind speed, and land cover (Figure 8).

Wind Speed showed a generally low and relatively stable response across much of the observed gradient, followed by a moderate increase at higher values. Human Modification exhibited an almost flat response curve, indicating comparatively limited variation in predicted suitability across the observed anthropogenic modification gradient.

The response curves for BIO4 (temperature seasonality) and Aspect were relatively flat, with only minor changes in predicted suitability across their respective environmental gradients. This pattern is consistent with their comparatively low variable-importance values in the ensemble model (Figure 8).

Overall, the response curves indicate that the predicted habitat suitability of *C. grande* is primarily associated with surface albedo, wind speed, land-cover characteristics, and slope, together with selected climatic variables such as BIO10 and BIO17. The combined patterns suggest that the potential distribution of the species is shaped by the interaction of climatic, topographic, surface, and landscape characteristics rather than by a single environmental factor.

#### 2.4.4. Potential Distribution and Habitat Extent

**Figure 9:**
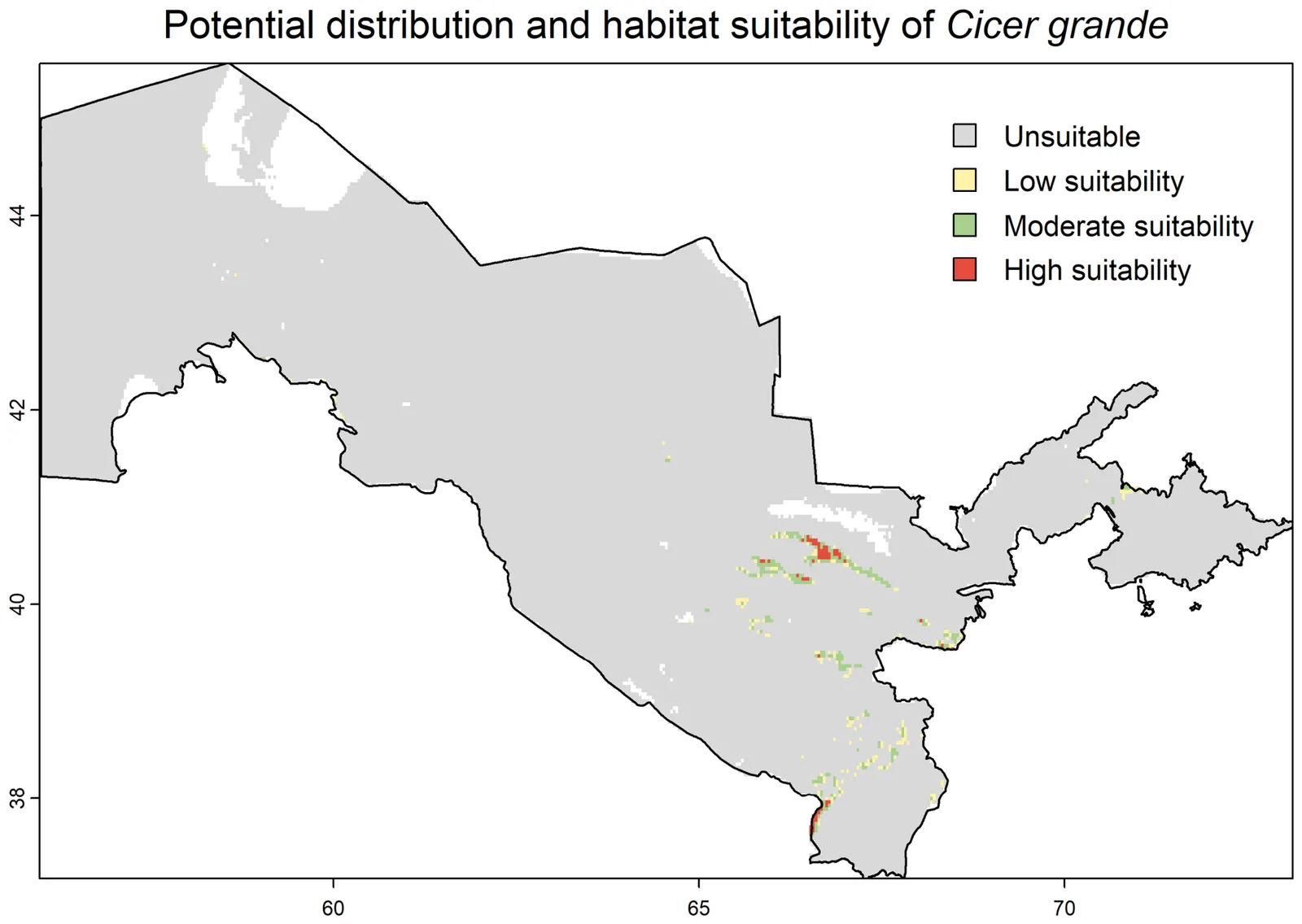
Potential distribution and habitat suitability of *C. grande* in Uzbekistan based on the TSS-based ensemble model. Habitat suitability was classified as unsuitable (0.00–0.20), low suitability (0.20–0.30), moderate suitability (0.30–0.60), and high suitability (0.60–1.00). White areas represent water bodies.

The ensemble species distribution model identified four habitat suitability classes for *C. grande* across Uzbekistan: unsuitable, low, moderate, and highly suitable habitats (Figure 9). The habitat suitability map indicated that environmentally suitable areas were predominantly concentrated within the mountainous regions of southern and central Uzbekistan.

Unsuitable habitats occupied the largest proportion of the study area, whereas highly suitable habitats were spatially restricted and mainly associated with the known distribution areas of *C. grande*. Moderate- and low-suitability habitats formed transitional zones surrounding the areas with higher predicted suitability.

The predicted distribution pattern was consistent with the known occurrence records of *C. grande*, particularly within the Nurata and Kuhitang mountain ranges. The highly suitable areas were relatively limited in extent, indicating that suitable environmental conditions for the species are spatially restricted.

These results highlight the geographically restricted ecological niche of *C. grande* and provide a spatial basis for identifying priority areas for field surveys, population monitoring, and conservation planning.

#### 2.4.5. Geographic Range and Preliminary IUCN Assessment

GeoCAT analysis of the 39 documented occurrence records estimated an extent of occurrence (EOO) of 5680.927 km^2^ and an area of occupancy (AOO) of 104 km^2^ for *C. grande* in Uzbekistan. When the two geographically disjunct mountain ranges were assessed separately, the Kuhitang Ridge had an EOO of 17.397 km^2^ and an AOO of 32 km^2^, whereas the Nurata Ridge had an EOO of 397.858 km^2^ and an AOO of 72 km^2^ (Table 3). The AOO of the species as a whole fall below the IUCN B2 threshold for Endangered (EN), while the EOO of the combined distribution falls within the Vulnerable (VU) threshold under B1. The much smaller EOO values obtained for the two individual mountain ranges indicate a highly restricted spatial distribution. However, these geographic-range thresholds alone do not constitute a definitive IUCN Red List category, because additional conditions under Criterion B must also be evaluated.

## 3. Discussion

### 3.1. Model Performance, Validation, and Predicted Distribution

This study employed a revised species distribution modelling (SDM) framework to assess the ecological niche and potential distribution of *C. grande* in Uzbekistan. The modelling approach incorporated a fixed calibration area with an independent evaluation dataset, alongside five-fold cross-validation within the calibration data, thereby providing a more rigorous assessment of predictive performance and reducing the risk of conflating internal validation with independent model testing.

Ensemble model results demonstrated generally strong predictive discrimination across algorithms. In five-fold cross-validation, MAXNET yielded the highest mean AUCroc (0.993 ± 0.007) and TSS (0.903 ± 0.128), whereas random forest (RF) and generalized boosted models (GBM) also showed robust performance. On the independent evaluation dataset, AUCroc values ranged from 0.952 ± 0.071 (GLM) to 0.999 ± 0.001 (RF), while TSS varied from 0.905 ± 0.143 (GLM) to 0.995 ± 0.001 (MAXNET). These metrics indicate that the ensemble approach maintains strong discriminatory power on held-out data, although performance differences among algorithms highlight the value of algorithm selection and multi-model inference.

Spatially, the predicted distribution of *C. grande* is strongly restricted within Uzbekistan, with suitable habitats predominantly concentrated in mountainous and foothill environments, overlapping substantially with known occurrence areas. This restricted pattern aligns with the ecological characteristics of narrow-range montane species, for which habitat availability is often constrained by the interplay of topography, surface conditions, climatic regimes, and land-cover attributes.

### 3.2. Environmental Drivers of Habitat Suitability

Variable importance analysis identified albedo as the most influential predictor of habitat suitability for *C. grande*, followed by wind speed, land cover, and slope. Among climatic variables, BIO17 (precipitation of driest quarter) and BIO10 (mean temperature of warmest quarter) showed moderate contributions, whereas land surface temperature (LST) and BIO4 (temperature seasonality) contributed comparatively less. This hierarchy suggests that the potential distribution of *C. grande* is shaped by a combination of surface reflectance characteristics, terrain structure, atmospheric conditions, and seasonal moisture–temperature regimes, rather than being governed predominantly by temperature alone.

The strong influence of albedo implies that surface reflectance—potentially integrating substrate properties, vegetation cover, and surface energy balance—may serve as an important proxy for suitable micro-environments, though this association should be interpreted as correlative rather than causal. Similarly, the substantial contribution of slope reinforces the species’ affinity for dissected montane terrain, while the role of wind speed may reflect local atmospheric and microclimatic conditions characteristic of exposed mountain and foothill settings.

Response curves further illustrated how predicted suitability shifts along environmental gradients, with pronounced changes observed for albedo, wind speed, land cover, and slope. Notably, although elevation was included in the initial variable set, it was not retained in the final predictor matrix after correlation- and variance-inflation-based selection. This exclusion does not imply ecological irrelevance; rather, the independent signal of elevation is likely attenuated by its strong collinearity with retained predictors such as BIO10, LST, and slope. Overall, these patterns underscore that climatic variables should be considered in conjunction with topographic and surface characteristics, rather than as isolated distributional determinants.

### 3.3. Restricted Distribution, Ecological Niche, and Comparison with Congeneric Taxa

The predicted distribution maps reveal that environmentally suitable habitats for *C. grande* are highly confined within Uzbekistan. Based on the revised TSS-weighted ensemble model, unsuitable habitats covered 423,049.70 km^2^, accounting for 98.51% of the modelled study area. Low-, moderate-, and highly suitable habitats occupied 2468.30 km^2^ (0.57%), 3141.26 km^2^ (0.73%), and 787.16 km^2^ (0.18%), respectively. Together, moderate- and highly suitable habitats amounted to only 3928.42 km^2^, or approximately 0.91% of the total modelled area. High-suitability areas were mainly concentrated in the Nurata and Kuhitang mountain systems, overlapping spatially with known occurrence records, and their extremely limited extent supports the interpretation that *C. grande* occupies a narrow ecological niche with a sharply restricted potential range.

This restricted montane distribution is consistent with patterns documented for many wild *Cicer* species across Central and Western Asia, which typically occur at elevations of approximately 1000–2500 m a.s.l. Such mid-elevation environments provide heterogeneous climatic and topographic gradients that may favour local adaptation while simultaneously limiting dispersal into adjacent lowlands. Comparable distributions have been reported for other endemic or narrowly distributed Fabaceae in the Pamir–Tian Shan region, reinforcing the biogeographical importance of montane landscapes for regional legume diversity [13]. In addition, the association of *C. grande* with Lithosols and Calcic Xerosols points to a relatively specific edaphic requirement. These shallow, skeletal, carbonate-rich substrates are typical of arid mountain slopes and may impose constraints on water and nutrient availability, contributing further to the ecological specialization and spatially restricted habitat suitability of the species.

### 3.4. Conservation Implications and Anthropogenic Considerations

Although human-modification variables contributed relatively little to the overall environmental variation in the SDM, this statistical result should not be interpreted as evidence that *C. grande* is immune to anthropogenic disturbance. Localized threats—including infrastructure development, livestock grazing, land-use change, and habitat degradation in montane areas—may affect small, fragmented populations even when broad-scale predictors show low relative importance. Given the geographically restricted nature of suitable habitats, even localized deterioration could have disproportionately large consequences for population persistence. Consequently, conservation assessments must integrate SDM outputs with field-based evaluations of population size, habitat condition, grazing intensity, and site-specific land-use pressures.

The conservation significance of *C. grande* is further underscored by its narrow potential distribution and limited suitable area. The GeoCAT analysis confirmed a restricted geographic range: although the combined extent of occurrence (EOO) for Uzbekistan was 5680.927 km^2^, EOO values calculated separately for the two disjunct mountain ranges were substantially smaller—397.858 km^2^ for the Nurata Ridge and merely 17.397 km^2^ for the Kuhitang Ridge. The combined area of occupancy (AOO) was 104 km^2^, with estimates of 72 km^2^ and 32 km^2^ for the Nurata and Kuhitang populations, respectively. These metrics highlight the highly localized distribution of the species and the conservation importance of both mountain systems. However, EOO and AOO thresholds alone are insufficient for assigning a definitive IUCN Red List category; additional Criterion B conditions—such as number of locations, subpopulation fragmentation, and evidence of continuing decline—must also be evaluated before a formal status can be proposed.

The highly suitable areas identified in this study provide a spatial basis for prioritizing targeted field surveys and population monitoring. Nevertheless, model predictions should be treated as screening tools rather than confirmed occurrence maps, and all priority sites require ground-truthing before being used for conservation boundary delineation. Furthermore, the present results should not be extrapolated as evidence of broad agricultural suitability. The SDM does not assess cultivation performance, productivity, physiological tolerance, or domestication potential; any future consideration of *C. grande* for cultivation would therefore require independent experimental studies addressing germination, growth, water requirements, soil adaptation, reproductive performance, and responses to managed environments.

### 3.5. Limitations and Future Research Directions

Several limitations of this study should be acknowledged. First, the SDM framework identifies correlative associations between environmental predictors and occurrence records, and although response curves illustrate patterns of predicted suitability, they do not provide direct evidence of physiological thresholds, competitive interactions, or causal ecological mechanisms. Second, the model is static and does not incorporate future climate scenarios, biotic interactions, or population dynamics; hence, statements regarding climatic sensitivity or potential range shifts remain speculative and require explicit hypothesis testing through experimental or long-term monitoring approaches. Third, while the independent validation demonstrated strong predictive discrimination, model extrapolation beyond the environmental space represented by the calibration data should be treated with caution, and the predicted suitability map is best interpreted as a relative index rather than an absolute probability of occurrence.

Future research should prioritize targeted field surveys to validate predicted high-suitability areas, particularly in under-sampled sectors of the Nurata and Kuhitang ranges, and should establish long-term population monitoring to assess demographic trends and regeneration status. Experimental studies are also needed to evaluate germination requirements, drought tolerance, edaphic preferences, and reproductive biology under both natural and ex situ conditions, especially if cultivation or ex situ conservation programmes are contemplated. Additionally, integrating genetic data with SDM outputs could clarify population structure, evolutionary history, and adaptive potential, thereby strengthening evidence-based conservation planning for this narrow-range endemic species.

## 4. Materials and Methods

### 4.1. Study Species and Occurrence Data

The study area is located in Uzbekistan, Central Asia, which places it within the broader arid and semi-arid mountain systems of the region [29]. Occurrence records of *C. grande* were compiled from field-based observations and the National Herbarium of Uzbekistan (TASH). Of the 39 initial occurrence records, 28 were obtained from field-based observations and documented occurrence localities, while 11 records were obtained from specimens deposited in TASH. During field surveys, geographic coordinates were recorded using a Global Positioning System (GPS) device. For historical herbarium records, geographic coordinates were obtained by georeferencing the locality information provided on the original specimens. The spatial precision of these historical records varied according to the level of geographic detail available in the original herbarium information and was therefore generally lower than that of the GPS-based field records.

**Figure 10:**
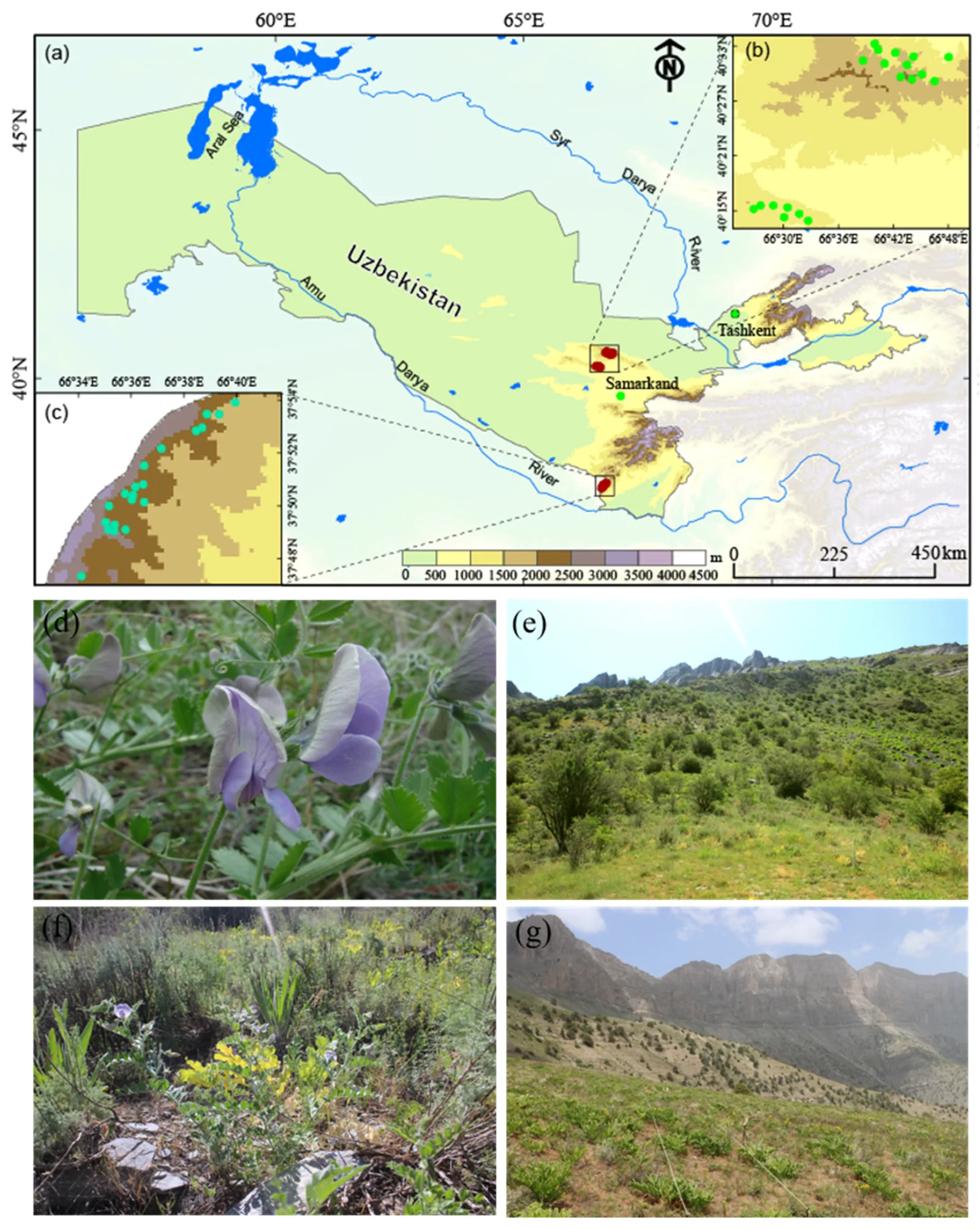
Location of the study area (**a**) in Uzbekistan. (**b**) Sampling sites of *C. grande* in the Nurata ridge. (**c**) Sampling sites of *C. grande* in the Kuhitang ridge. (**d**) General view of *C. grande* in the Nurata ridge. (**e**) Plant communities in the Nurata ridge. (**f**) General view of *C. grande* in the Kuhitang ridge. (**g**) Plant communities in the Kuhitang ridge.

The geographic locations of the study area and documented occurrence sites of *C. grande* in the Nurata and Kuhitang ridges are shown in Figure 10.

To minimize spatial autocorrelation and sampling bias within the occurrence dataset, spatial thinning was applied using the spThin package in R. A minimum nearest-neighbour distance of 1 km was employed to remove spatially clustered records. Following the thinning procedure, 31 spatially independent occurrence records were retained for subsequent modelling analyses.

Because reliable absence data were unavailable for *C. grande*, pseudo-absence points were generated using the random sampling algorithm implemented in the BIOMOD2 package. Three independent sets of 1000 pseudo-absence points each were randomly generated from areas where the species had not been recorded. The modelling procedure consisted of three independent datasets, each containing the 31 presence records and 1000 pseudo-absence points. Thus, each modelling replicate comprised 1031 records, while 3000 pseudo-absence points were generated across the three replicates.

From July 2022 to July 2024, geobotanical surveys were carried out on plant communities containing *C. grande* across two local cenopopulations: LP1 on the Nurata Ridge and LP2 on the Kuhitang Ridge. Within each cenopopulation, 10 m × 1 m transects were established in both perpendicular directions and subdivided into 1 m^2^ quadrats. In each quadrat, the floristic composition and projective cover of co-occurring species were recorded. Cover was estimated as the percentage of ground surface covered by the vertical projection of aboveground plant parts, following the Braun–Blanquet approach. The ontogenetic structure of *C. grande* local cenopopulations was assessed following the established approaches of Rabotnov and Uranov. Individuals were classified according to their morphological and developmental characteristics into the following ontogenetic states: juvenile (j), immature (im), virginile (v), young generative (g_1_), middle generative (g_2_), old generative (g_3_), subsenile (ss), and senile (s). The relative proportions of these ontogenetic states were used to construct the ontogenetic spectrum of each local cenopopulation.

Cenopopulation classification was performed according to the approaches proposed by A.A. Uranov and O.V. Smirnova, together with the Δ–ω (delta–omega) classification system of L.A. Zhivotovsky. The Δ index was used to characterize the age structure of the cenopopulation, whereas the ω index was used to characterize its efficiency. Based on the Δ–ω classification, the studied cenopopulations were assigned to the corresponding age-state categories.

### 4.2. Species Distribution Modelling

**Figure 11:**
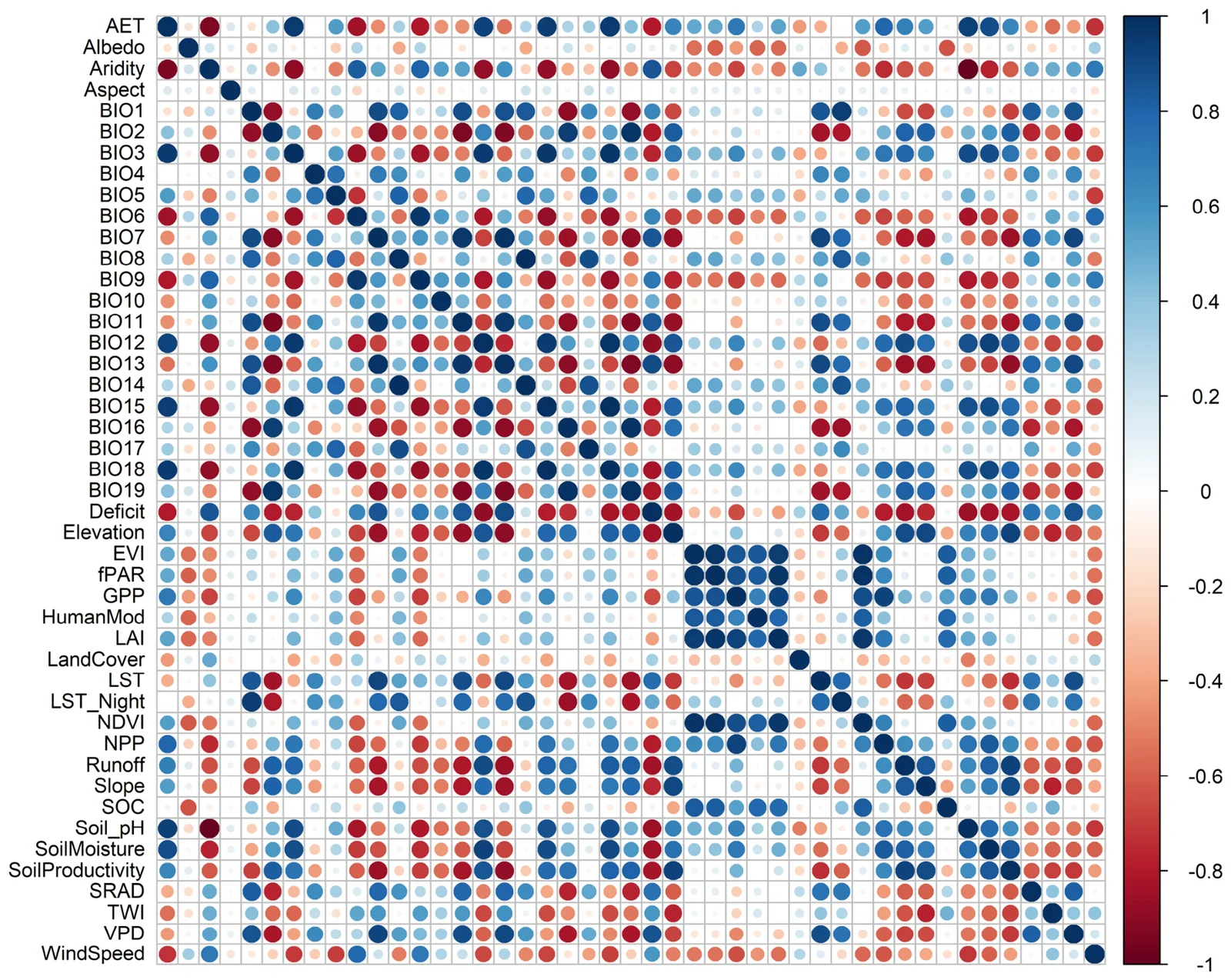
Correlation matrix of the 45 environmental variables included in the initial dataset and used for variable selection. Blue indicates positive correlations and red indicates negative correlations; colour intensity and circle size indicate the strength of the correlation (ranging from −1 to +1). The complete list of variable abbreviations, full names, and definitions is provided in Appendix A Table A1.

Species distribution modelling was performed using the BIOMOD2 framework based on the georeferenced occurrence records of *C. grande* and the selected environmental predictors. An initial set of 45 environmental variables was evaluated, from which 10 predictors—Albedo, Aspect, BIO4, BIO10, BIO17, Human Modification (HumanMod), Land Cover, Land Surface Temperature (LST), Slope, and Wind Speed—were retained for the final modelling analysis. Because reliable absence data were unavailable, three independent sets of 1000 pseudo-absence records were generated using the random pseudo-absence strategy. To distinguish internal cross-validation from independent model evaluation, the occurrence dataset was divided once into an 80% calibration subset and a 20% independent testing subset. The calibration data were used for model development and five-fold cross-validation, whereas the independent testing data were withheld from model fitting and cross-validation and were used only for final assessment of predictive performance. Five algorithms were evaluated, including generalized linear models (GLM), generalized additive models (GAM), generalized boosted models (GBM), random forest (RF), and MAXNET. Model performance was assessed using the Area Under the Receiver Operating Characteristic Curve (AUC) and the True Skill Statistic (TSS). Ensemble predictions were generated using the mean ensemble approach (EMmean), with model selection based on validation performance rather than the independent testing data. The relative importance of environmental predictors was assessed using permutation-based variable importance, and response curves were generated to characterize the predicted response of *C. grande* to individual environmental gradients. Final ensemble predictions were projected across Uzbekistan and expressed as habitat-suitability probabilities ranging from 0 to 1. These predictions were subsequently classified into four suitability categories: unsuitable (0.00–0.20), low suitability (0.20–0.30), moderate suitability (0.30–0.60), and high suitability (0.60–1.00) (Figure 11).

### 4.3. Ecological Niche Modeling

Ecological niche modelling for *C. grande* was conducted in R using the BIOMOD2 package, with spatially filtered presence records and randomly generated pseudo-absences. Ten environmental predictors (Albedo, Aspect, BIO4, BIO10, BIO17, Human Modification Index, Land Cover, Land Surface Temperature, Slope, and Wind Speed) were standardized to 1 km resolution and a common coordinate system. Five algorithms—Generalized Linear Model (GLM), Generalized Additive Model (GAM), Generalized Boosting Model (GBM), Random Forest (RF), and MAXNET—were implemented.

The occurrence data were partitioned into a fixed calibration subset (80%) and an independent evaluation subset (20%). Five-fold cross-validation was performed within the calibration subset for model training, while the withheld 20% served exclusively for final independent evaluation. Model performance was assessed using the Area Under the Receiver Operating Characteristic Curve (AUC) and the True Skill Statistic (TSS). In addition, a principal component analysis (PCA) was carried out on environmental values at occurrence localities as a separate exploratory step, independent of predictor selection for modelling.

To reduce algorithm-specific uncertainty, an ensemble model was generated by weighted averaging of individual algorithm predictions, with weights based on TSS scores. The resulting ensemble output yielded a continuous suitability map (range 0–1), which was reclassified into four categories: unsuitable (0.00–0.20), low (0.20–0.30), moderate (0.30–0.60), and high (0.60–1.00) suitability for subsequent spatial analyses.

### 4.4. Mapping and Visualization

Geographic distribution patterns and elevational range of *C. grande* within Uzbekistan were visualized using ArcGIS version 10.8.2. Species distribution maps were developed based on a 30 m resolution digital elevation model derived from the Shuttle Radar Topography Mission (SRTM), allowing detailed representation of topographic variation and elevation-dependent distribution patterns.

### 4.5. IUCN-Based Geographic Range Assessment

The potential extinction risk of *C. grande* was evaluated following the IUCN Red List Categories and Criteria (Version 3.1) and relevant guidelines [30]. The extent of occurrence (EOO) and area of occupancy (AOO) were estimated using (Geospatial Conservation Assessment Tool, RBG Kew); based on the 39 documented occurrence records. EOO was calculated as the minimum convex polygon encompassing the occurrence records, while AOO was estimated using a 2 × 2 km grid cell [31]. To characterize the spatial extent of the species within its two geographically disjunct mountain ranges, EOO and AOO were additionally calculated separately for the Nurata Ridge (19 records) and Kuhitang Ridge (20 records). The resulting values were compared with the geographic-range thresholds of IUCN Criterion B1 (EOO) and B2 (AOO).

## 5. Conclusions

This study provides an integrated assessment of the ecological niche and potential distribution of the endemic *C. grande* in Uzbekistan, using a revised species distribution modelling framework that combined five-fold cross-validation within the calibration dataset with an independent evaluation dataset to ensure robust predictive performance. The results indicate that the potential distribution of *C. grande* is highly restricted, predominantly confined to mountainous and foothill environments. Albedo emerged as the most influential predictor, followed by wind speed, land cover, and slope, while climatic variables (BIO17 and BIO10) contributed moderately. These findings suggest that the species’ distribution is shaped by a combination of surface, topographic, climatic, and landscape attributes, rather than being governed solely by temperature.

Quantitative estimates from the TSS-weighted ensemble model showed that 98.51% of the study area was classified as unsuitable, with low, moderate, and high-suitability habitats accounting for 0.57%, 0.73%, and 0.18%, respectively. Together, moderate- and high-suitability areas occupied only about 0.91% of the modelled region. This extremely narrow extent of potentially suitable environments, together with the limited Extent of Occurrence and Area of Occupancy, underscores the conservation importance of the Nurata and Kuhitang mountain systems. The resulting suitability map provides a useful spatial framework for prioritizing field surveys, population monitoring, and conservation planning; however, modelled suitability should be treated as a screening tool to identify priority areas rather than as a substitute for ground-based verification.

Given the narrow ecological niche of *C. grande*, the present results should not be interpreted as evidence of broad agricultural potential. Future research should integrate targeted field validation, long-term population monitoring, physiological experiments, and ex-situ cultivation trials to clarify the species’ environmental tolerances, regeneration constraints, and adaptive capacity. Such multidisciplinary efforts will be essential for improving ecological understanding and supporting effective long-term conservation strategies for this narrow-range endemic species.

## Figures and Tables

**Table 1:** Floristic composition of local cenopopulations.

Species Name	Life Form	Family	LP1	LP2
*Crataegus songarica* K. Koch	Tree	Rosaceae	+	−
*Juniperus seravschanica* Kom.	Tree	Cupressaceae	−	+
*Cotoneaster soongoricus* (Regel & Herder) Popov	Shrub	Rosaceae	+	−
*Lonicera bracteolaris* Boiss. & Buhse	Shrub	Caprifoliaceae	+	−
*Lonicera nummulariifolia* Jaub. & Spach	Shrub	Caprifoliaceae	5	+
*Astragalus bactrianus* Fisch.	Shrub	Fabaceae	−	+
*Cerasus amygdaliflora* Nevski	Shrub	Rosaceae	−	+
*Rosa canina* L.	Shrub	Rosaceae	−	+
*Artemisia tenuisecta* Nevski	Semi shrub	Asteraceae	5	−
*Ziziphora clinopodioides* Lam.	Semi shrub	Lamiaceae	+	−
*Acantholimon majewianum* Regel	Semi shrub	Plumbaginaceae	−	+
*Allium jodanthum* Vved.	Perennial	Amaryllidaceae	+	−
*Astragalus farctissimus* Lipsky	Perennial	Fabaceae	+	−
*Astragalus sewertzowii* Bunge	Perennial	Fabaceae	+	−
*Carex pachystylis* J. Gay	Perennial	Cyperaceae	−	+
*Cicer grande* (Popov) Korotkova	Perennial	Fabaceae	25	8
*Delphinium batalinii* Huth	Perennial	Ranunculaceae	+	−
*Eremurus kaufmannii* Regel	Perennial	Asphodelaceae	−	+
*Eremurus turkestanicus* Regel	Perennial	Asphodelaceae	+	−
*Euphorbia glomerulans* (Prokh.) Prokh.	Perennial	Euphorbiaceae	−	+
*Ferula kokanica* Regel & Schmalh.	Perennial	Apiaceae	+	−
*Ferula kuhistanica* Korovin	Perennial	Apiaceae	−	12
*Festuca valesiaca* Schleich. ex Gaudin	Perennial	Poaceae	+	−
*Galium pamiroalaicum* Pobed.	Perennial	Rubiaceae	+	−
*Gentiana olivieri* Griseb.	Perennial	Apiaceae	−	+
*Geranium transversale* (Kar. & Kir.) Vved. ex Pavlov	Perennial	Geraniaceae	+	−
*Haplophyllum acutifolium* (DC.) G. Don	Perennial	Rutaceae	−	+
*Hedysarum mogianicum* (B. Fedtsch.) B. Fedtsch.	Perennial	Fabaceae	10	−
*Hordeum bulbosum* L.	Perennial	Poaceae	−	+
*Hypericum scabrum* L.	Perennial	Hypericaceae	+	+
*Leontice ewersmannii* Bunge	Perennial	Berberidaceae	−	+
*Linaria popovii* Kuprian.	Perennial	Plantaginaceae	+	−
*Lindelofia macrostyla* (Bunge) Popov	Perennial	Boraginaceae	+	−
*Lophanthus schtschurowskianus* Regel	Perennial	Lamiaceae	+	−
*Nepeta olgae* Regel	Perennial	Lamiaceae	−	+
*Onopordum leptolepis* DC.	Perennial	Asteraceae	−	+
*Origanum tyttanthum* Gontsch.	Perennial	Lamiaceae	+	−
*Pedicularis olgae* Regel	Perennial	Orobanchaceae	+	−
*Poa bulbosa* L.	Perennial	Poaceae	20	+
*Pseudopodospermum hissaricum* (C. Winkl.) Zaika, Sukhor. & N. Kilian	Perennial	Asteraceae	−	+
*Ranunculus paucidentatus* Schrenk	Perennial	Ranunculaceae	+	−
*Rheum maximowiczii* Losinsk.	Perennial	Polygonaceae	−	+
*Silene paranadena* Bondarenko & Vved.	Perennial	Caryophyllaceae	+	−
*Stipa caucasica* Schmalh.	Perennial	Poaceae	−	+
*Taraxacum monochlamydeum* Hand.-Mazz.	Perennial	Asteraceae	−	+
*Taraxacum officinale* F.H. Wigg.	Perennial	Asteraceae	−	+
*Tulipa affinis* Botschantz.	Perennial	Liliaceae	+	−
*Ungernia oligostroma* Popov & Vved.	Perennial	Amaryllidaceae	+	−
*Valeriana ficariifolia* Boiss.	Perennial	Caprifoliaceae	+	−
*Cousinia radians* Bunge	Biennial	Asteraceae	+	−
*Cirsium vulgare* (Savi) Ten.	Biennial	Asteraceae	−	+
*Cousinia platylepis* Fisch., C.A. Mey. & Avé-Lall.	Biennial	Asteraceae	−	+
*Tragopogon coelesyriacus* Boiss.	Biennial	Asteraceae	−	+
*Verbascum songaricum* Schrenk ex Fisch. & C.A. Mey.	Biennial	Scrophulariaceae	−	+
*Bromus lanceolatus* Roth	Annual	Poaceae	5	−
*Noccaea perfoliata* (L.) Al-Shehbaz	Annual	Brassicaceae	+	−

Note: “+” indicates the presence of a species, whereas “−” indicates its absence.

**Table 2:** Biometric and Demographic Characteristics of Populations.

	Parameter	LP1	LP2
Biometric Characteristics	Total height (cm)	60–63	43–45
	Aboveground height (cm)	45–48	28–32
	Root length (cm)	15–16	15–13
	Root diameter (cm)	2–2.5	0.8–1.2
	Leaf length (cm)	0.7–2.3	0.6–1.5
	Leaf width (cm)	0.3–0.7	0.3–0.6
Demographic parameter	I_r_	1.06	0.95
	I_a_	0.03	0.02
	I_e_	9.92	24
	Individual density (1 m^2^)	1.6	2
	P ecol (1 m^2^)	1.88	2.66
	Total number	32	40
	∆	0.23	0.60
	ω	0.30	0.60
Population type		Mature	Young

Notes: I_r_—regeneration coefficient, I_a_—aging index, I_e_—efficiency index, P ecol—ecological density, Δ—delta, ω—omega.

**Table 3:** CR, EN, and VU indicate the IUCN geographic-range thresholds corresponding to the calculated EOO and AOO values and should not be interpreted as definitive Red List categories without evaluation of the additional conditions of Criterion B.

Geographic Unit	Occurrence Records	EOO (km^2^)	EOO Threshold Category	AOO (km^2^)	AOO Threshold Category
Nurata Ridge	19	397.858	EN	72	EN
Kuhitang Ridge	20	17.397	CR	32	EN
Uzbekistan (all records)	39	5680.927	VU	104	EN

## Data Availability

The datasets generated during and/or analyzed during the current study are available from the corresponding author upon reasonable request.

## Appendix A

**Table A1:** Complete list and definitions of the 45 environmental variables included in the initial dataset.

№	Abbreviation	Full Name/Variable	Definition
1	AET	Actual Evapotranspiration	Actual amount of water transferred from land to atmosphere through evaporation and transpiration
2	Albedo	Surface Albedo	Fraction of incoming solar radiation reflected by the surface
3	Aridity	Aridity Index	Measure of climatic dryness based on precipitation and atmospheric water demand
4	Aspect	Aspect	Direction of the terrain slope
5	BIO1	Annual Mean Temperature	Mean annual temperature
6	BIO2	Mean Diurnal Range	Mean monthly temperature range averaged over the year
7	BIO3	Isothermality	Ratio of mean diurnal temperature range to annual temperature range
8	BIO4	Temperature Seasonality	Variation in temperature throughout the year
9	BIO5	Max Temperature of Warmest Month	Maximum temperature of the warmest month
10	BIO6	Min Temperature of Coldest Month	Minimum temperature of the coldest month
11	BIO7	Temperature Annual Range	Difference between BIO5 and BIO6
12	BIO8	Mean Temperature of Wettest Quarter	Mean temperature during the wettest quarter
13	BIO9	Mean Temperature of Driest Quarter	Mean temperature during the driest quarter
14	BIO10	Mean Temperature of Warmest Quarter	Mean temperature during the warmest quarter
15	BIO11	Mean Temperature of Coldest Quarter	Mean temperature during the coldest quarter
16	BIO12	Annual Precipitation	Total annual precipitation
17	BIO13	Precipitation of Wettest Month	Total precipitation of the wettest month
18	BIO14	Precipitation of Driest Month	Total precipitation of the driest month
19	BIO15	Precipitation Seasonality	Variation in precipitation throughout the year
20	BIO16	Precipitation of Wettest Quarter	Total precipitation during the wettest quarter
21	BIO17	Precipitation of Driest Quarter	Total precipitation during the driest quarter
22	BIO18	Precipitation of Warmest Quarter	Total precipitation during the warmest quarter
23	BIO19	Precipitation of Coldest Quarter	Total precipitation during the coldest quarter
24	Elevation	Elevation	Height above sea level
25	EVI	Enhanced Vegetation Index	Satellite-derived vegetation greenness index
26	FVC	Fractional Vegetation Cover	Proportion of ground covered by vegetation
27	GPP	Gross Primary Productivity	Total carbon fixation by vegetation through photosynthesis
28	HumanMod	Human Modification Index	Degree of anthropogenic modification of natural environments
29	LAI	Leaf Area Index	Total leaf area per unit ground area
30	LandCover	Land Cover	Type and spatial distribution of land-cover classes
31	LST	Land Surface Temperature	Temperature of the land surface during daytime
32	LST_Night	Night-time Land Surface Temperature	Land surface temperature during nighttime
33	NDVI	Normalized Difference Vegetation Index	Satellite-derived indicator of vegetation density and greenness
34	NPP	Net Primary Productivity	Net carbon accumulation by vegetation after plant respiration
35	Runoff	Surface Runoff	Amount of precipitation flowing over the land surface
36	Slope	Slope	Steepness of the terrain
37	SOC	Soil Organic Carbon	Amount of organic carbon stored in soil
38	Soil_pH	Soil pH	Measure of soil acidity or alkalinity
39	SoilMoisture	Soil Moisture	Amount of water contained in the soil
40	SoilProductivity	Soil Productivity	Indicator of the capacity of soil to support plant growth
41	SRAD	Solar Radiation	Amount of incoming solar radiation
42	SWC	Soil Water Content	Amount or proportion of water contained in soil
43	TWI	Topographic Wetness Index	Topographic indicator of potential soil moisture accumulation
44	VPD	Vapor Pressure Deficit	Atmospheric moisture deficit indicating evaporative demand
45	WindSpeed	Wind Speed	Speed of near-surface wind
